# OM-FBA: Integrate Transcriptomics Data with Flux Balance Analysis to Decipher the Cell Metabolism

**DOI:** 10.1371/journal.pone.0154188

**Published:** 2016-04-21

**Authors:** Weihua Guo, Xueyang Feng

**Affiliations:** Department of Biological Systems Engineering, Virginia Polytechnic Institute and State University, Blacksburg, Virginia, United States of America; University of Nebraska Medical Center, UNITED STATES

## Abstract

Constraint-based metabolic modeling such as flux balance analysis (FBA) has been widely used to simulate cell metabolism. Thanks to its simplicity and flexibility, numerous algorithms have been developed based on FBA and successfully predicted the phenotypes of various biological systems. However, their phenotype predictions may not always be accurate in FBA because of using the objective function that is assumed for cell metabolism. To overcome this challenge, we have developed a novel computational framework, namely omFBA, to integrate multi-omics data (e.g. transcriptomics) into FBA to obtain omics-guided objective functions with high accuracy. In general, we first collected transcriptomics data and phenotype data from published database (e.g. GEO database) for different microorganisms such as *Saccharomyces cerevisiae*. We then developed a “Phenotype Match” algorithm to derive an objective function for FBA that could lead to the most accurate estimation of the known phenotype (e.g. ethanol yield). The derived objective function was next correlated with the transcriptomics data via regression analysis to generate the omics-guided objective function, which was next used to accurately simulate cell metabolism at unknown conditions. We have applied omFBA in studying sugar metabolism of *S*. *cerevisiae* and found that the ethanol yield could be accurately predicted in most of the cases tested (>80%) by using transcriptomics data alone, and revealed valuable metabolic insights such as the dynamics of flux ratios. Overall, omFBA presents a novel platform to potentially integrate multi-omics data simultaneously and could be incorporated with other FBA-derived tools by replacing the arbitrary objective function with the omics-guided objective functions.

## Introduction

**Fig 1:**
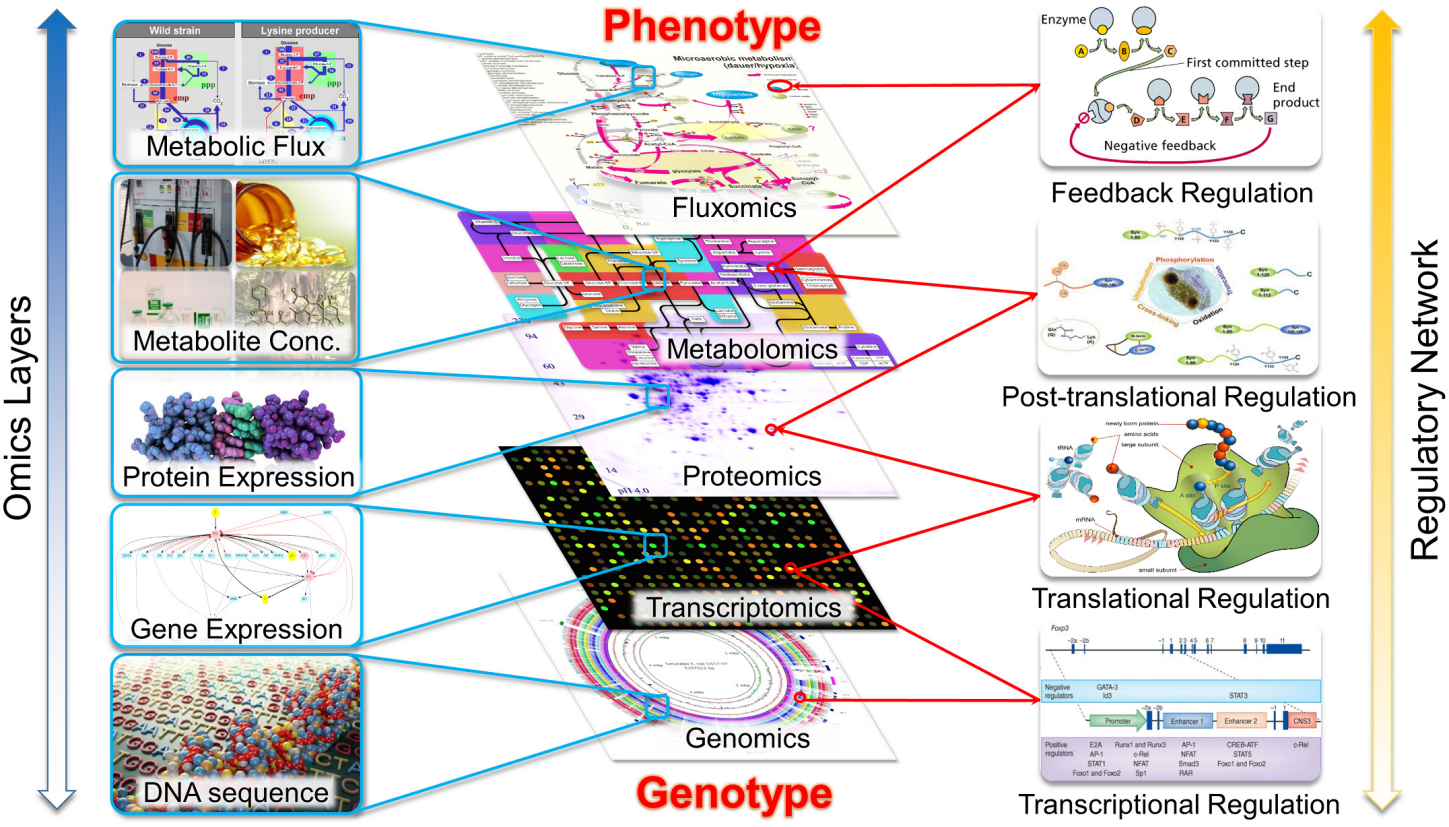
Complex interactions of various components in cell metabolism. Multi-omics data has provided the quantitative readouts of these components, which helps us to elucidate the interactions among the multi-layer regulations.

Cell metabolism is regulated over multiple levels with the participation of various types of cell components[1], e.g., gene expression via transcription process and the protein synthesis via translation and post-translational modification (Fig 1), which mystifies genotype-phenotype correlations. Since the phenotype is the net result of these interactions, it is immensely important to unveil the different cell components and their interactions, not only for an integrated understanding of physiology, but also for the practical applications of biological systems as cell factories[2–4]. High-throughput omics data provides quantitative readouts of these cell components, including the cell’s DNA sequence (i.e., genomics[5, 6]), mRNA expression (i.e., transcriptomics[7]), metabolite abundance (i.e., metabolomics[8, 9]), protein composition (i.e., proteomics[10–12]), and *in vivo* enzyme activities (i.e., fluxomics[13, 14]). This valuable biological information enables the identification and quantification of individual components of a biological system, and we are now facing the challenge of understanding the interactions among these components[1, 15] by appropriately analyzing and interpreting the omics data.

Metabolic modeling is the computational approach widely used for modeling complex metabolic networks and predicting cell phenotype based on the stoichiometric constraints of metabolic reactions. One of the most commonly used approaches is flux balance analysis (FBA)[16], in which a genome-scale metabolic model is supplied in the form of a stoichiometric matrix that conveys the molecularity of each metabolite in each reaction. This is then followed by the identification of an arbitrary objective function to optimize, due to the underdetermined FBA system. Since the early 1990s, FBA has been widely used to simulate cell phenotypes with tremendous success because of its genome-scale estimation and fast computation speed [16–18]. However, the arbitrary objective function remains problematic and sometimes leads to inaccurate phenotype predictions [16, 19]. To integrate the FBA approach with the omics data, several FBA-derived algorithms have been developed for phenotype prediction with one or two types of omics data. These algorithms can be broadly classified into two categories [20, 21]: 1) the switch approach (e.g., GIMME[22] and iMAT[23]), which turns reaction fluxes on or off based on threshold gene expression levels, and 2) the valve approach (e.g., E-Flux[24] and PROM[25]), which regulates reaction fluxes based on relative gene/protein expressions. One of the fundamental limitations for all of these approaches is that all make the underlying assumption that gene transcription is linearly correlated with the flux of the reactions that they encode[26]. This correlation has been found to be inaccurate by many physiological studies of cells [21, 27, 28]. In addition, most of these algorithms can integrate transcriptomics only or transcriptomics and proteomics only [27, 29]. To our best knowledge, no algorithm can simultaneously integrate multi-omics data for phenotype prediction so far. Therefore, to intently overcome the ill-defined assumptions of the genotype-phenotype correlation and to potentially integrate multi-omics data for phenotype prediction, we develop a novel FBA-derived algorithm, omFBA, to correlate the genotype, e.g., transcriptomics data, with the phenotype, e.g., growth rate and product yields, by using an omics-guided objective function.

**Fig 2:**
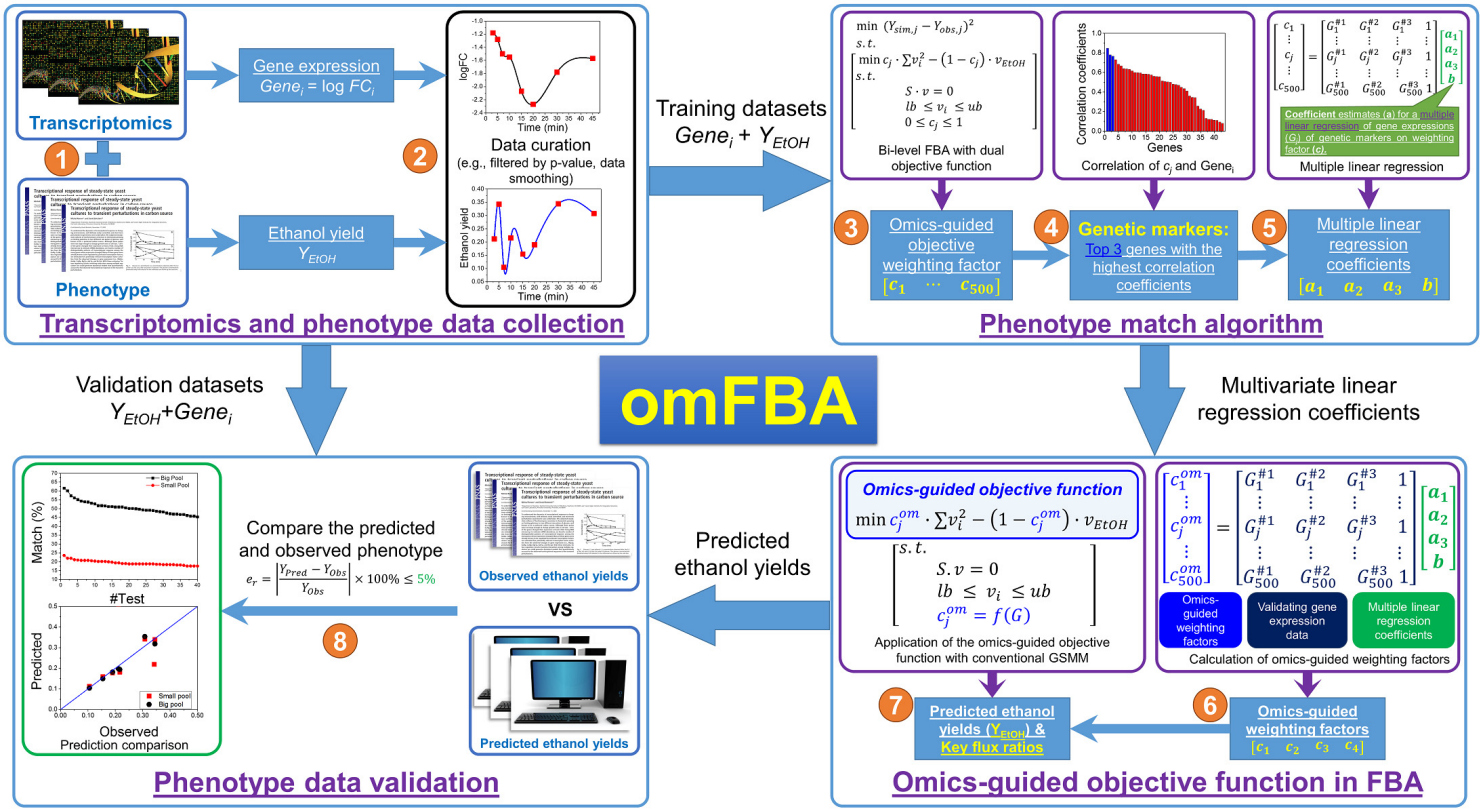
Scheme of omFBA algorithm. Four modules are designed to implement omFBA algorithm: 1) transcriptomic-phenotype data collection (Step 1~2), 2) “phenotype match” algorithm (Step 3~5), 3) omics-guided objective function in FBA (Step 6~7), and 4) phenotype data validation (Step 8).

In general, we implemented a proof-of-concept study for this novel algorithm, omFBA, to accurately predict the phenotype of a model eukaryotic microorganism, *Saccharomyces cerevisiae*, by integrating the transcriptomics data with FBA via the omics-guided objective function (Fig 2). We first collected the transcriptomics data, i.e., exponential fold changes of gene expression levels, and the corresponding phenotype data, i.e., ethanol yields, from the GEO database and previous publication [30]. We randomly separated the datasets into two equal parts: one part was used to develop the omics-guided objective function; and the other part was used to validate the omics-guided objective function by applying the transcriptomics data to predict cell phenotypes and comparing them with the observed phenotypes. We found that omFBA accurately predicted the ethanol yields in most of the cases tested (>80%) and provided valuable insights of yeast metabolism such as the key flux ratios that were consistent with previous ^13^C-MFA studies [31–33]. In sum, the novel algorithm we developed in this study, omFBA, could accurately predict cell phenotypes, and more importantly, provide in-depth understanding of the interactions between transcriptomics and phenotypes, which could be extended for various cell components to help achieve better understanding of cell metabolism.

## Results

### Overview of omFBA algorithm

To develop and validate omFBA algorithm as a novel approach to integrate transcriptomics data with FBA via omics-guided objective function, we have designed a computational platform with four modules: 1) transcriptomics-phenotype data collection, 2) “phenotype match” algorithm, 3) omics-guided objective function in FBA, and 4) phenotype data validation. In brief, in Module 1, we collected the transcriptomics-phenotype correlated data from a published study[30], curated the data based on *p*-value, smoothed the data, and randomly separated all the datasets into two equal parts, i.e., training and validation datasets, for the development and validation of omFBA algorithm, respectively. In Module 2, we developed the “phenotype match” algorithm by first using a dual objective function, with unknown weighting factors assigned to minimizing overall enzyme usage and maximizing ethanol yield, to simulate the cell phenotype; then searched for the “phenotype matched” weighting factors that could lead to the best fitting of cell phenotypes in the training datasets; and finally, quantitatively correlated these “phenotype matched” weighting factors with the transcriptomics data from the training datasets via multivariate regression. Next in Module 3, we applied such empirical correlation between the transcriptomics data and the “phenotype matched” weighting factors in the dual objective function in FBA, together with the transcriptomics data from validation datasets, to derive the omics-guided objective function. We then used this omics-guided objective function in the genome-scale metabolic model of *S*. *cerevisiae* to predict the ethanol yields and to provide in-depth biological insights, e.g., key flux ratios. In Module 4, we compared our predicted ethanol yields that were derived from transcriptomics data with the observed ones in the validation datasets for evaluation of the prediction accuracy of omFBA algorithm. We also validated the biological insights implicated by the omFBA algorithm by comparing them with the experimental discoveries from ^13^C-metabolic flux analysis (^13^C-MFA). To ensure our algorithm is statistically reliable, we have repeated the Module 1~4 for 40 times by randomly assigning different training and validation datasets. The detailed development of each module was shown in the following sections.

### Module 1: transcriptomics-phenotype data collection

We have collected the transcriptomics-phenotype correlated data from a published study[30], in which Ronen and Botstein studied transcriptomics responses of *S*. *cerevisiae* for the substrate shift from the unfavorable galactose to favorable glucose at low and high concentrations (i.e., 0.2 g/L and 2g/L, respectively) at different time points. The transcriptomics responses were measured by microarray and represented by the fold changes of gene expression levels of all the detectable genes (>6300 genes) after the substrate shift. We collected all these data from the GEO database (GSE4158) as the “big” gene pool for omFBA algorithm by running the GEO2R web tool [34]. Comparing to this “big” gene pool, a “small” gene pool was also defined by Ronen and Botstein [30], who selected a small set of genes that could play deciding roles in determining the yeast metabolic responses to substrate shift. We also extracted transcriptomics data of the same genes from the paper as the “small” gene pool to implement the omFBA algorithm for evaluating the impacts of transcriptomics data on the omFBA algorithm. The glucose-based ethanol yields were selected as phenotype in this study, since no other phenotype but ethanol yield was provided in the experimental data (S2 Table).

The *p*-values of all transcriptomics data were also collected to reflect the data quality. To filter the low-quality data, we selected the cutoff *p*-value as 0.95, the same cutoff value used in the published paper [30]. That is to say, we only used the genes that had differentiated expression level with *p*<0.95 to develop omFBA. The corresponding phenotype data, i.e., ethanol yields, was collected and calculated from the Fig 1 of the published paper, which showed the fermentation profiles after the substrate shift. We found only 8 time points were available with *p*-value-filtered transcriptomics data and the corresponding phenotype data, which captured the dynamics of transcriptional responses and phenotype changes but were not enough for developing omFBA algorithm. We have used a prebuilt function namely ‘csaps’ in MATLAB to smooth the kinetic profile of gene expression data and phenotype data, based on the raw data of gene expression and phenotype at the 8 time points. In general, we used cubic smoothing spline to provide a smoothed curve with 1000 points to capture the shape of the temporal gene expression and phenotype. In this case, we could overcome the limitation of lacking gene expression data for training our OM-FBA algorithm while still maintaining the high fidelity of the kinetic profile of gene expression and phenotype. We randomly chose 500 datasets as the training datasets while the remaining 500 datasets were used for model validation.

### Module 2: “phenotype match” algorithm

**Fig 3:**
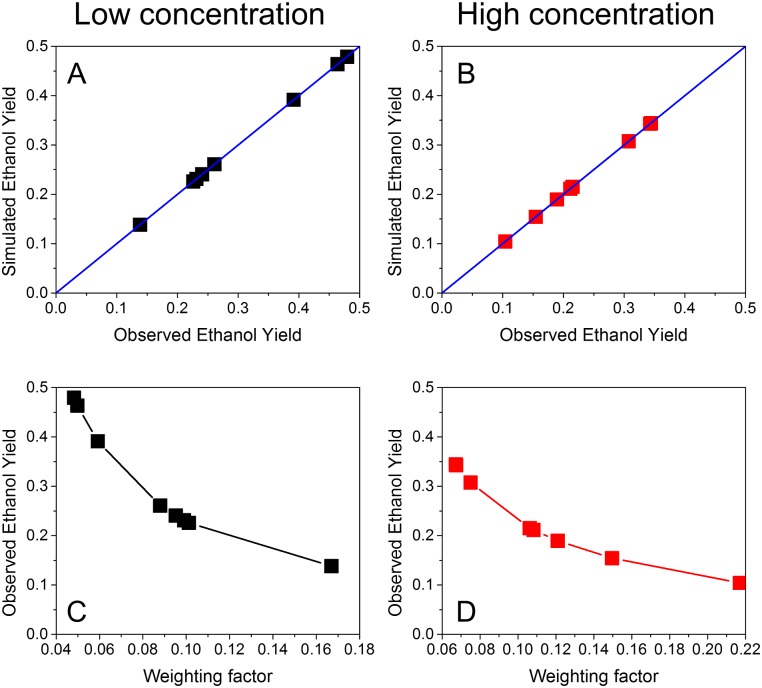
“Phenotype match” algorithm for low and high glucose conditions. The simulated and observed ethanol yields matched well for low (A) and high (B) glucose conditions, respectively. Negative correlations between the weighting factors of minimizing the overall enzyme usage and the observed ethanol yield were found for low (C) and high (D) glucose conditions.

Using the training datasets developed in Module 1, we developed the “phenotype match” algorithm as a bi-level FBA algorithm to find the optimal objective function that leads to accurate simulations of the observed phenotypes. To achieve this, we first selected a dual objective function that included two items: minimizing overall enzyme usage (i.e., $\text{min}\;\sum v_{i}^{2}$) and maximizing ethanol yield (i.e., max *v*_*EtOH*_), indicating the trade-off between the overall enzyme activities and the ethanol production. The similar dual objective function was previously applied to successfully predict growth kinetics of *Shewanella oneidensis* [35]. The weighting factors of these two items were unknown. However, by fine-tuning the weighting factors, we could match the phenotype predicted by FBA with the observed phenotype very well. As shown in Fig 3, a good fitting was observed from the carbon metabolism shift in both low and high glucose conditions, which proved that it is feasible for the “phenotype match” algorithm to find the suitable objective functions in FBA. In addition, we correlated the observed ethanol yields with the phenotype-matched weighting factors in front of minimizing overall enzyme usage for both low and high glucose conditions, and found that a negative correlation was discovered between the weighting factors and ethanol yields. In general, when the weighting factor of minimizing overall enzyme usage increased, the ethanol production decreased, which was consistent with trade-off between the overall enzyme activities and the ethanol production. We found the fluxes towards biomass formation were zero when using the bi-level optimization for minimizing enzyme usage and maximizing ethanol production. This is consistent with the experimental data since no significant change was found in cell number and size (10%) during the glucose pulse experiments [30]. In comparison, we have also implemented the FBA by applying two commonly used objective functions: maximizing the growth rate, and maximizing the ethanol yield (S1 Fig). The simulated ethanol yield was 0.11 g ethanol/g glucose when maximizing the growth rate, which underestimated the ethanol production in the real experiments. The simulated ethanol yield was 0.51 g ethanol/g glucose when maximizing the ethanol yield, which overestimated the ethanol production in the real experiments.

**Fig 4:**
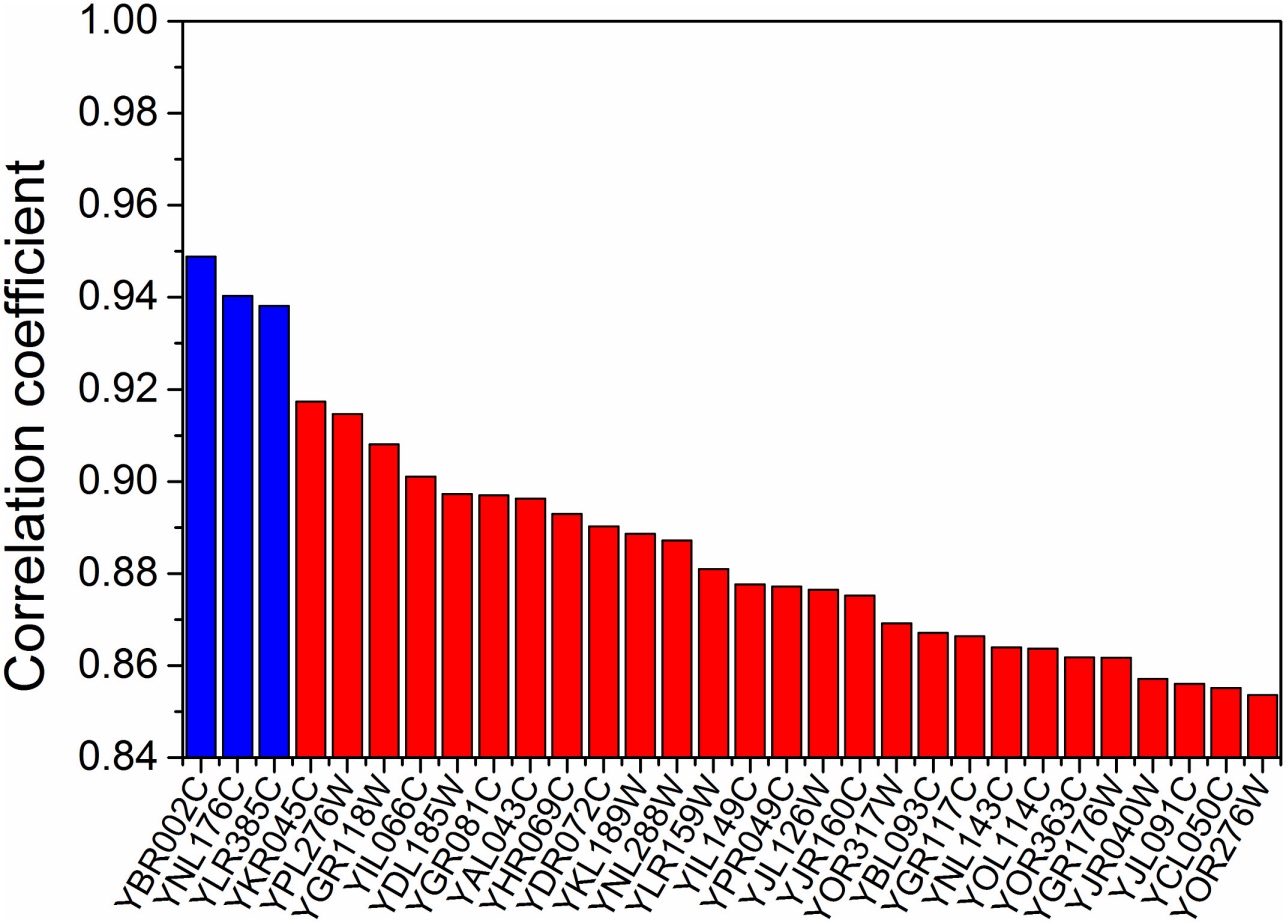
Correlation between phenotype-matched weighting factors and gene expressions. The absolute values of the correlation coefficients in one of the training datasets were ranked from high to low (only the top 30 genes were shown here). The top 3 genes were chosen as the genetic markers to derive the omics-guided objective function (blue bars).

With this appropriately selected dual-objective function and the phenotype-matched weighting factors, we next calculated the correlation coefficients between these weighting factors and the transcriptomics data for each gene in both the “big pool” and the “small pool”, respectively. We ranked all of the genes based on the correlation coefficients, and picked the top 3 genes with the highest absolute values of the correlation coefficients as the genetic markers (Fig 4). We then applied the multiple linear regression approach to quantitatively correlate the phenotype-matched weighting factors and the expression levels of the genetic markers. The regression equation, which represents the quantitative correlation of the transcriptomics and the phenotype, was calculated and used next in Module 3 to derive the omics-guided objective function.

### Module 3 and 4: application and validation of omics-guided objective function

With the regression equation derived in Module 2 to connect the expression levels of the genetic markers and the weighting factors used in the dual objective function of FBA, we could use the transcriptomics data in the validation datasets to derive the so-called “omics-guided objective function” for phenotype prediction. In general, we collected the expression levels of the genetic markers in each of the validation datasets, applied the regression equation to derive the weighting factors for the dual objective function, and then used the dual objective function in a well-established genome-scale metabolic model, iND750 [36, 37], to predict the ethanol yields.

**Fig 5:**
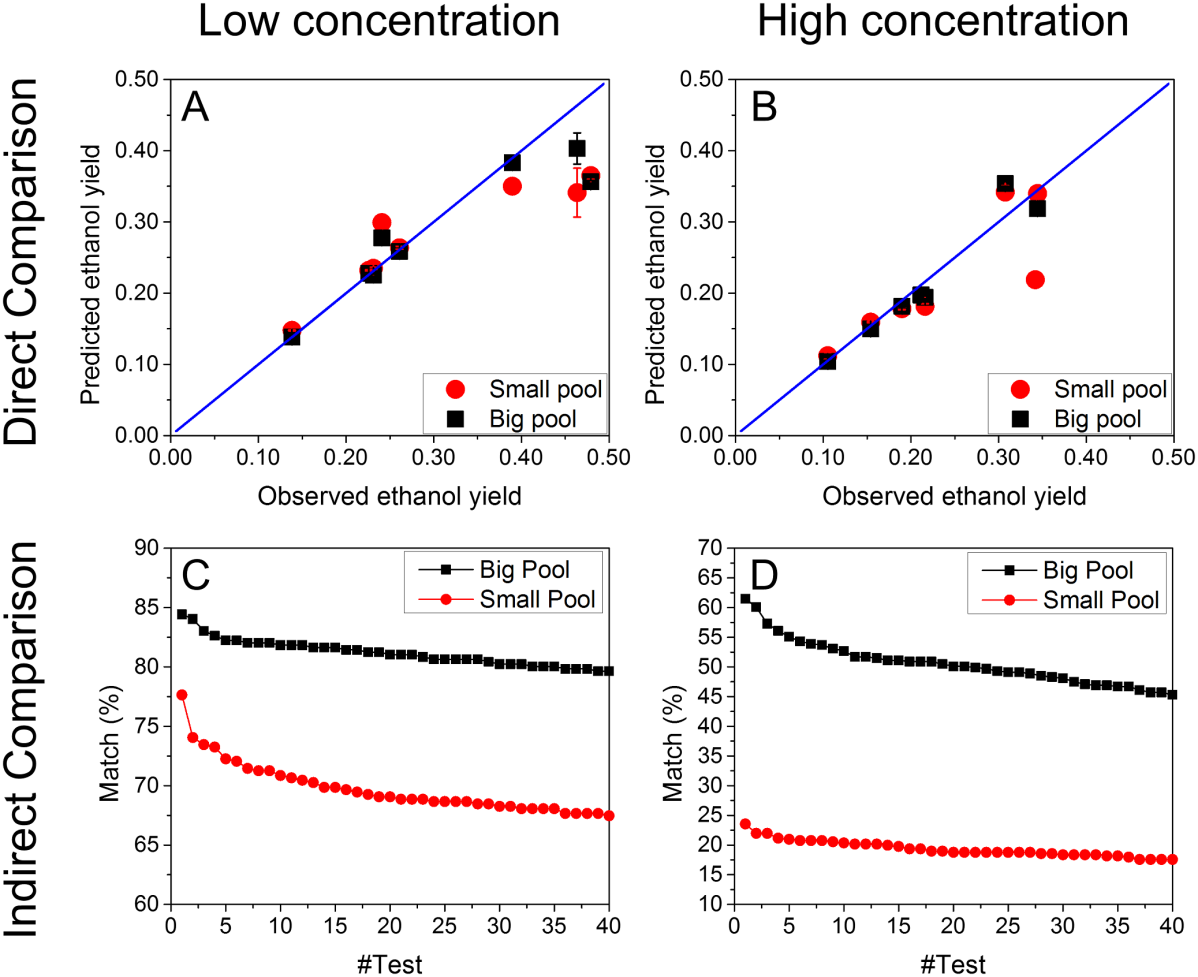
Prediction accuracy of omFBA algorithm. Direct comparison of the predicted and observed ethanol yields in low (A) and high (B) glucose conditions. The omFBA algorithm was repeated for 40 times and the percentage of matched predictions of omFBA algorithm were calculated and ranked for low (C) and high (D) glucose conditions.

We derived the omics-guided objective function for both the “big pool” and “small pool” of genes, respectively, in both low and high glucose conditions. For each of the 500 validation datasets, we checked the predicted ethanol yield and the observed ones. As mentioned previously, we randomly assigned the 1000 datasets into 500 training datasets and 500 validation datasets, and repeated this procedure for 40 times to make sure our predictions were statistically reliable. As shown in Fig 5, we found that for all of the 40 rounds of predictions, we could obtain 80~85% (Fig 5C) and 47~63% (Fig 5D) match (i.e., <5% difference) between the predicted and observed ethanol yields when using the “big pool” of genes for deriving the omics-guided objective function to simulate glucose metabolism at low and high concentrations, respectively. When using the “small pool” of genes, 67~77% (Fig 5C) and 20~25% (Fig 5D) match (i.e., <5% difference) between the predicted and observed ethanol yields were observed for glucose metabolism at low and high concentrations, respectively. In addition, we also plotted the raw data of ethanol yields (i.e., the 8 datasets that were originally collected from Ronen and Botstein ‘s work) versus the predicted ones in low (Fig 5A) and high (Fig 5B) glucose conditions and found that using either “big pool” or “small pool” of genes could lead to good fittings (R^2^>0.80). It is also worth noting that the prediction accuracy for the low and high glucose condition was different. While at least 65% match with the experimental data could be achieved at low glucose condition, only 60% match at best could be achieved at high glucose condition. We have examined the reason for the difference of the prediction between low and high concentration data. We found that at the low glucose condition, ~10% of the genes had strong correlations with phenotype-matched weighting factors with the absolute value of the correlation coefficient > 0.9 (S2 Fig). However, at the high glucose condition, <1% of the genes had strong correlations with phenotype-matched weighting factors with the absolute value of the correlation coefficient > 0.9. Therefore, the poor correlation between gene expression and the phenotype-matched weighting factors could account for the lower match at the high glucose condition.

**Fig 6:**
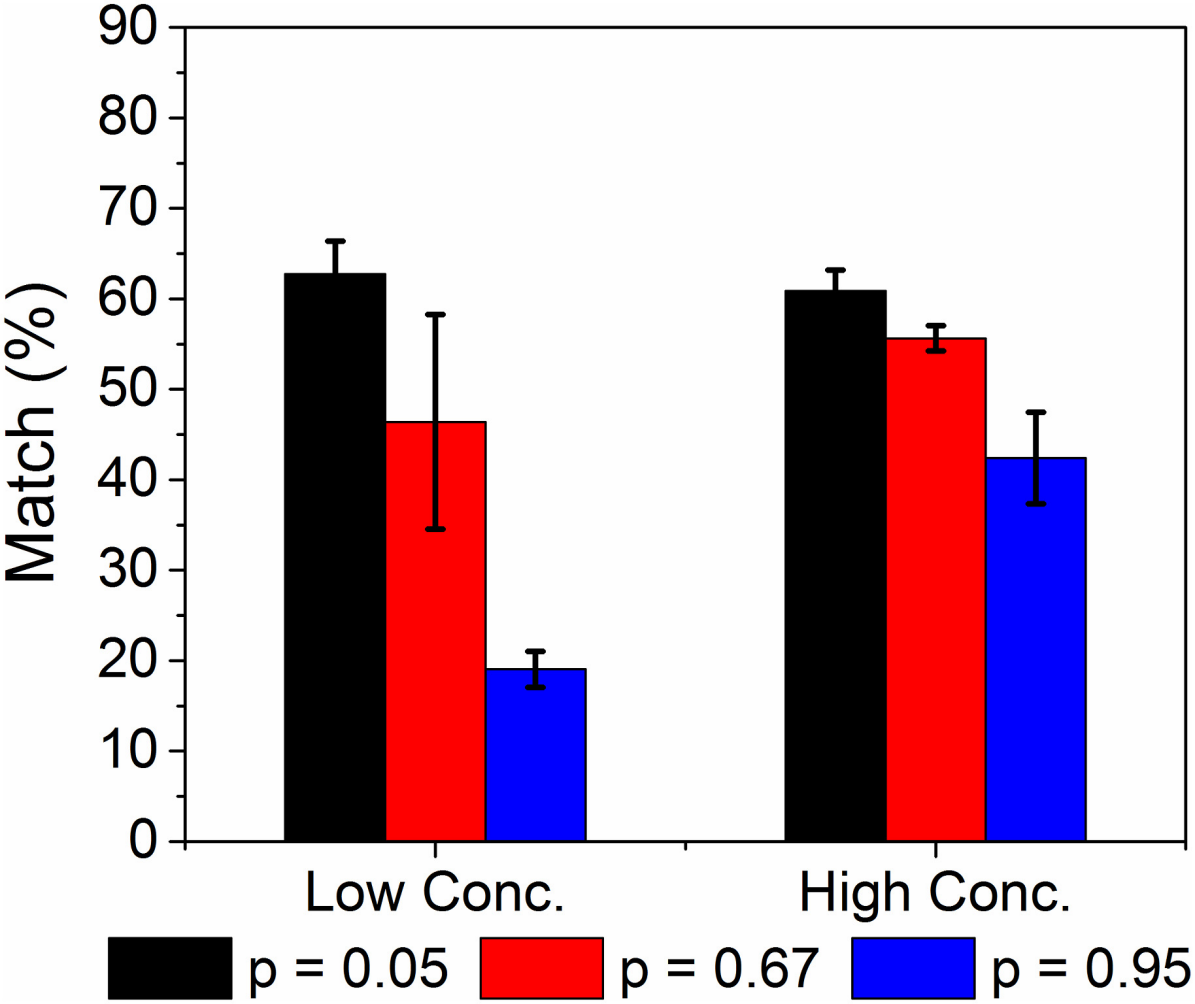
Effect of cutoff *p*-value on omFBA prediction using “small pool” of genes. Three cutoff *p*-values, i.e., 0.05, 0.67, and 0.95, were used to filter the transcriptomics data. For each cutoff *p*-value, we re-ran the omFBA algorithm for 40 times and calculated the percentage of matches between the predicted and the observed ethanol yields in the validation datasets.

Since the accuracy of omFBA was not satisfactory (<60% matches) when using “small pool” of genes to predict yeast metabolism in low and high glucose condition, we next tried to investigate the factors that could affect the omFBA prediction. One factor that could potentially affect the omFBA prediction is the noisy gene expressions, which is notoriously known by biologists for decades [38–41]. To examine the impact of the variability of transcriptomics data on the omFBA algorithm, we chose three different cutoff *p*-values, i.e., 0.05, 0.67, and 0.95, to represent different variability levels of the transcriptomics data for the genes in the “small pool”. By filtering the transcriptomics data using the selected cutoff *p*-values, we re-ran omFBA for 40 times and compared the prediction accuracy in *p* = 0.05, 0.67 and 0.95, respectively. As shown in Fig 6, the prediction accuracy for ethanol yields under both low and high glucose conditions dramatically increased when we chose a smaller cutoff *p*-value, with >60% ethanol yields in the validation datasets could be well matched (<5% difference) with the observed ethanol yields at *p* = 0.05. This clearly suggested that the quality of transcriptomics data could be a deciding factor for accurate prediction of cell metabolism when using omFBA. With the breakthroughs in high-throughput, high-accuracy analytical methods for omics analysis, we envision the demand for high-quality omics data would be well met in very near future.

**Fig 7:**
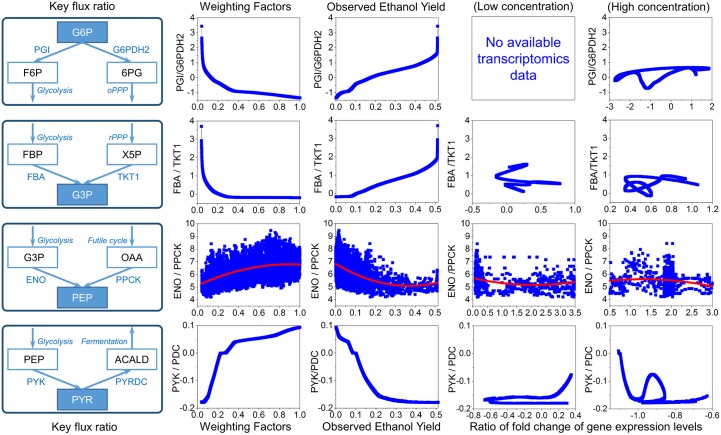
Key flux ratio analysis. Four key flux ratios (PGI/G6PDH2, FBA/TKT1, ENO/PPCK, and PYK/PDC) were selected to be correlated with phenotype-matched weighting factors, observed ethanol yields, and the ratios of the corresponding gene expression levels for low and high glucose condition. All the values of the ratios were exponential. Abbreviations: PGI, glucose-6-phosphate isomerase; G6PDH2, glucose 6-phosphate dehydrogenase; FBA, fructose-bisphosphate aldolase; TKT1, transketolase; ENO, enolase; PPCK, phosphoenolpyruvate carboxykinase; PYK, pyruvate kinase; PYRDC, pyruvate decarboxylase.

Overall, omFBA could predict the ethanol yields solely from the transcriptomics data via the development of “omics-guided objective function”. It is interesting to notice that using “big pool” of genes led to much better accuracy than using “small pool” of genes, indicating that there were still many unknown regulons in *S*. *cerevisiae* that played pivotal roles in controlling yeast metabolism but have not yet been fully studied. In addition to the accurate predictions of the phenotypes, omFBA could also provide valuable biological insights, e.g., dynamics of flux ratios, to unravel the intracellular metabolic rewiring. Here in this study, we selected and calculated four key flux ratios, namely PGI/G6PDH2, FBA/TKT1, ENO/PPCK, and PYK/PDC, which controlled the flux distribution in the central metabolism (Fig 7). To study the key metabolic responses to the substrate shift in *S*. *cerevisiae*, we have correlated these key flux ratios with the “phenotype matched” weighting factors, observed ethanol yields, and the ratios of corresponding gene expressions. We found clear trends between the key flux ratio and the ethanol production. In general, when the ethanol production was increased, the ratios of glycolysis to the pentose phosphate pathways were decreased, while the ratios of glycolysis to the futile cycle and the fermentation pathway were increased. Such observations were consistent with the previous ^13^C-MFA studies on yeast metabolism [32, 33, 42] (Table 1). However, no correlations between the flux ratios and gene expression ratios were observed for either low or high glucose concentration. Such poor correlation between gene expression and metabolic fluxes has been proved by previous reports [31], and emphasized the merit of developing novel algorithm like omFBA that does not rely on the assumption of correlated gene expression and metabolic fluxes. Since the growth rates from ^13^C-MFA studies were not exactly the same as what we observed in this study because of the different experimental set-up, e.g., culture mode (batch culture or chemostate) and growth condition (medium, sugar concentration), we did not directly compare the growth rate between our studies and the ^13^C-MFA studies.

**Table 1:** Key flux ratios compared with previous studies using ^13^C metabolic flux analysis.

Flux ratio^a^	Correlation with increased ethanol yields		Corresponding genes	Correlation with gene expression ratios	
	omFBA	Previous studies [32, 33, 42]		omFBA	Previous studies[31]
**PGI/G6PDH2**	Positive	Positive	YBR196C/YNL241C	No correlation^b^	No correlation
**FBA/TKT1**	Positive	Positive	YKL060C/YPR074C	No correlation	No correlation
**ENO/PPCK**	Negative	Negative	YGR254W/YKR097W	No correlation	No correlation
**PYK/PYRDC**	Negative	Negative	YAL038W/YLR044C	No correlation	No correlation

PGI, glucose-6-phosphate isomerase; G6PDH2, glucose 6-phosphate dehydrogenase; FBA, fructose-bisphosphate aldolase; TKT1, transketolase; ENO, enolase; PPCK, phosphoenolpyruvate carboxykinase; PYK, pyruvate kinase; PYRDC, pyruvate decarboxylase.

^a^All the abbreviations were from the iND750 model.

^b^No correlation was defined as the cases in which the correlation coefficient (absolute value) was smaller than 0.2.

## Discussion

### Comparison between omFBA algorithm and the “Big Data” regression approach

With the abundance of transcriptomics-phenotype datasets, a natural question one may ask is how good (or bad) is omFBA when compared to regression approach using the “big data”. In brief, we could also design an algorithm using the “big data” to directly correlate the ethanol yields and the transcriptomics data in the training datasets and apply the regression equation to predict the ethanol yields solely from the transcriptomics data in the validation datasets, similarly as we did for omFBA. In fact, we have implemented such “Big Data” regression approach (S1 Dataset and S3 Fig) and found that the ethanol yields could indeed be accurately predicted (e.g., 82–90% matches when using “big pool” of genes in low glucose concentration, and 80–85% matches when using “big pool” of genes in high glucose concentration, S4 Fig). Comparing omFBA to the “Big Data” regression approach, we found that the prediction accuracy of omFBA was similarly good. When comparing the genetic markers used by omFBA and the “Big Data” regression approach (S1 Table), we indeed found some genes were used as the genetic markers in both approaches (S5 Fig), which could explain the good fittings generated from both approaches. However, one distinct advantage of omFBA compared to “Big Data” approach is the capability to provide valuable and reliable biological insights, such as the dynamics of flux ratios as shown in Fig 7. Rather than simply fitting the cell phenotypes, omFBA also provides a way to investigate the complex gene-flux-phenotype cross talking by developing the omics-guided objective functions. Such information is particularly useful since it could decipher the interactions of various cell components and rationally guides the metabolic engineering of organisms.

In this study, we chose the top 3 genes whose expression levels were most correlated with the phenotype-matched weighting factors as our important factors. Because little is known about how exactly the genetic markers influenced the phenotype-matched weighting factors, it is possible that some genetic markers, which played important roles in affecting phenotype-matched weighting factors but had expression levels not well correlated with the phenotype-matched weighting factors, could be left out (i.e., omitted-variable bias). However, we would like to emphasize that purpose of this proof-of-concept study is not to develop a “perfect” model to completely decipher the mechanism of how gene expression and cell phenotype are linked. Instead, this study offered a novel route to explore the possibility of using transcriptomics data to predict some of the metabolic behaviors (e.g., ethanol production).

### Prerequisites of omFBA algorithm

It is worth noting that one of the prerequisites for developing omFBA algorithm is the abundant, curated, and correlated omics-phenotype datasets, which, unfortunately, are not often available. Currently, many of the database constructed only collected one type of omics data, e.g., Gene Expression Omnibus (GEO)[34, 43–45], the European Bioinformatics Institute (EBI)[46, 47], and Many Microbe Microarrays Database (M3D)[48] for collecting gene expression data; and from the Proteomics DB[49] for collecting proteomics data. Although thousands of datasets are enabled for users to query for omics analysis (e.g., transcriptomics analysis), these datasets cannot provide the details about the phenotype such as cell growth rate, and hence, have limited applications in elucidating the correlations between transcriptomics and phenotype of microorganisms. To overcome this challenge, we have done some preliminary studies to construct the correlated omics-phenotype database, namely integrated Transcriptomic And Phenotype (iTAP) database [50], to collect the transcriptomics-phenotype correlated data. Despite the fact that the phenotype data was never reported in a standardized format and the curation of correlated transcriptomics–phenotype datasets was extremely tedious and time-consuming, till now, we have successfully correlated 57 and 143 datasets of transcriptomics and phenotype for *E*. *coli* and *S*. *cerevisiae*, respectively. As the first of its kind, the iTAP database was suitable for omFBA to provide sufficient data and allow the direct phenotype prediction from the transcriptomics analysis. We are planning to use more datasets from the iTAP database that we constructed to apply omFBA. However, one challenge in extending omFBA to other studies is the incompleteness of the meta-data. Basically, when we constructed the iTAP database, we found that many of the studies only reported the data that was most interested to the authors, e.g., growth rates alone, and left out the rest of the valuable information such as glucose consumption rates and ethanol production rates. The paper published by Ronen and Botstein was so far the most complete meta-data we could find. Therefore, we used this database to prove the concept of omFBA. It is also worth mentioning that while only transcriptomics data was used in this study, the platform of omFBA can be easily extended to include all types of omics data because of using the regression analysis to derived omics-guided objective function. For example, when using the multi-omics datasets (e.g., transcriptomics, proteomics, metabolomics datasets), we can follow the similar flowchart of omFBA in this study to rank the top cell components, e.g., gene expression, protein synthesis, metabolite concentration, that are highly correlated with the weighing factors in the “phenotype matched” objective function, and then used such connection to simulate cell phenotypes.

In summary, we have developed a novel FBA-derived algorithm, omFBA, to integrate the transcriptomics data into FBA via the omics-guided objective function for the accurate prediction of phenotypes and the in-depth simulation of biological insights. Compared to the “Big Data” regression approach, omFBA could achieve the similarly good predication but is superior in uncovering novel biological insights such as dynamics of metabolic flux ratios. The quality of transcriptomics data was found to be an important factor that affected the omFBA prediction. Although the transcriptomics-phenotype correlated datasets required by omFBA are still limiting, we envision that such challenge could be overcome by efforts such as the construction of iTAP database. With the correlated omics-phenotype data, omFBA algorithm could be a powerful approach to link genotype and phenotype and unravel the mysteries of cell metabolism.

## Methods and Models

### Transcriptomics and phenotype data collection

To develop the omFBA algorithm, we have selected a published paper with the transcriptomics-phenotype correlated data [30]. In the paper, Ronen and Botstein studied transcriptomics responses (i.e., the fold changes of gene expression levels) of *S*. *cerevisiae* at different time points after the shift of the substrates in chemostat, namely, from the unfavorable galactose to favorable glucose in low and high concentrations (i.e., 0.2 g/L and 2 g/L, respectively).

The transcriptomics data, i.e., the exponential fold changes of gene expression levels with *p*-values, was obtained by running the GEO2R web tool [34] with the control group set as T = 0 h (i.e., the starting time for the substrate shift). These transcriptomics data for all the time points have been collected as the “big” gene pool for the development of omFBA. In addition, the transcriptional analysis by Ronen and Botstein [30] indicated that a small set of genes could play deciding roles in determining the yeast metabolic responses to substrate shift. Therefore, we also extracted transcriptomics data of the same genes from the paper as a “small” gene pool, which could examine the impact of the pool size of the transcriptomics data on the omFBA. The *p*-value of the transcriptomics data tested the statistical significance level of the fold changes and was used to quantify the data quality. In the original published paper, the author selected 0.95 as the *p*-value cutoff value to remove the low-quality data [30]. To develop the omFBA algorithm, we followed the same cutoff value to filter the transcriptomics data in low quality by ignoring this gene in the database.

The phenotype data, i.e., ethanol yield (w/w), has been calculated based on the glucose and ethanol concentrations for the whole time courses, i.e., from 0 to 240 minutes, extracted from figures of the original paper. We have ignored the time points in the stationary phase of both low and high concentration datasets during the development of omFBA algorithm.

After removing the time points in stationary phase, we found only 8 time points were available with *p*-value-filtered transcriptomics data and recalculated phenotype data, which captured the dynamics of transcriptional responses and phenotype changes but were not enough for developing omFBA algorithm. We then smoothed both transcriptomics and phenotype data at a linear space including 1000 points by using the prebuilt function, i.e., “*csaps*”, in MATLAB (Step 2, Fig 2). Based on these 1000 points, we randomly selected 500 points as the training dataset for the development of omFBA algorithm. The other 500 points have been used to validate and evaluate the omFBA algorithm.

### Phenotype match algorithm

We developed the “phenotype match” algorithm to fine-tune FBA for accurate prediction of the phenotype data with a selected dual objective function in a bi-level FBA algorithm. The dual objective function includes two items: minimizing the overall enzyme usage and maximizing the ethanol yield, which reflects the trade-off between the overall enzyme activities and the ethanol production. The following mathematical equation was used to represent the dual objective function:

$$\text{min}c_{j}\sum v_{i}^{2}-\left(1-c_{j}\right)v_{EtOH}$$ (1)

where, *v*_*i*_ was all the fluxes in the genome-scale metabolic model; *v*_EtOH_ was the flux of the ethanol exchange; *c*_j_ was the phenotype-matched weighting factor for data point *j* of training dataset. This dual-objective function has been applied to a bi-level FBA algorithm as the inner objective function:

$$\begin{array}{l} \text{min}\;{\left(Y_{sim,j}-Y_{obs,j}\right)}^{2}\\ s.t.\\ \left[\begin{array}{c} \text{min}c_{j}\cdot \sum v_{i}^{2}-\left(1-c_{j}\right)\cdot v_{EtOH}\\ s.t.\\ S\cdot v\;=\;0\\ lb\;\leq v_{i}\;\leq ub \end{array}\right]\\ 0\leq c_{j}\leq 1 \end{array}$$ (2)

where, *Y*_sim,j_ and *Y*_sim,j_ were the simulated and observed ethanol yields (*w*/*w*) from inner FBA problem and training dataset, respectively. The outer objective function in this algorithm was minimizing the variance between the simulated ethanol yields and the observed ethanol yields from the training dataset by tuning the weighting factor of the dual-objective function. The stoichiometric matrix and boundary conditions were derived from the BiGG database [37], iND750[36], a genome-scale metabolic model of *S*. *cerevisiae*. To evaluate whether or not different metabolic models would affect the phenotype-matched weighting factors, we chose another genome-scale metabolic model of *S*. *cerevisiae* (iMM904 [51]), and re-ran our phenotype-match algorithm. We found that the weighting factors derived from the original model (i.e., iND750) and the new model (i.e., iMM904) were highly correlated (S6 Fig), with R^2^>0.99. Also, both model used almost the same gene-protein-pathway mapping. Therefore, we concluded that the network reconstruction would not significantly, if not at all, affect the omFBA. The inner FBA problem, as a quadratic optimization problem, was solved by the prebuilt solver, “*quadprog*”, in MATLAB. The outer optimization problem was solved by the “grid search” algorithm by using an in-house MATLAB algorithm with step-wise search in a range of the weighting factor ([0, 1.00]) with 10^−4^ for each step.

Next, we calculated the correlation coefficients between the phenotype-matched weighting factors and the transcriptomics data for each gene. We selected the top 3 genes with the highest absolute values of correlation coefficients as the genetic markers. To prevent over-fitting the data, we applied F-test (one-tail with cutoff *p*-value = 0.10) [52] to determine if we should accept (or reject) a new genetic marker in the linear regression model that used the “genetic markers” to fit phenotype-matched weighting factors. We found that when using top 3 instead of top 2 “genetic markers” in the linear regression model, the fitting became significantly improved, e.g., *p*-value was 0.09 (< cutoff *p*-value) for low concentration glucose condition using big gene pool. In other word, the introduction of the top 3 “genetic markers” was necessary to improve the model fitting and the data was not over-fitted.

We then quantitatively correlated the phenotype-matched weighting factors and the expression levels of the genetic markers using the multiple linear regression approach in MATLAB (“*regress*” command), which was shown in eq 3:

$$\left[\begin{array}{c} c_{1}\\ \vdots \\ c_{j}\\ \vdots \\ c_{500} \end{array}\right]=\left[\begin{array}{cccc} G_{t1}^{\#1} & G_{t1}^{\#2} & G_{t1}^{\#3} & 1\\ \vdots & \vdots & \vdots & \vdots \\ G_{tj}^{\#1} & G_{tj}^{\#2} & G_{tj}^{\#3} & 1\\ \vdots & \vdots & \vdots & \vdots \\ G_{t500}^{\#1} & G_{t500}^{\#2} & G_{t500}^{\#3}1 & 1 \end{array}\right]\;\left[\begin{array}{c} a_{1}\\ a_{2}\\ a_{3}\\ b \end{array}\right]$$ (3)

where, *c*_j_ was the phenotype-matched weighting factors of training dataset *j*; $G_{tj}^{\#1},\;G_{tj}^{\#2},\;G_{tj}^{\#3}$ were the expression levels of the three genetic markers in training dataset *j*, respectively; and *a*_1_, *a*_2_, *a*_3_, *b* were the regression coefficients for the genetic markers and the linear part, respectively.

### Omics-guided objective function in FBA

With the regression equation derived from the “phenotype match” algorithm, we then used the transcriptomics data from the validation datasets to derive the omics-guided weighting factors:

$$\left[\begin{array}{c} c_{1}^{om}\\ \vdots \\ c_{j}^{om}\\ \vdots \\ c_{500}^{om} \end{array}\right]=\left[\begin{array}{cccc} G_{v1}^{\#1} & G_{v1}^{\#2} & G_{v1}^{\#3} & 1\\ \vdots & \vdots & \vdots & \vdots \\ G_{vj}^{\#1} & G_{vj}^{\#2} & G_{vj}^{\#3} & 1\\ \vdots & \vdots & \vdots & \vdots \\ G_{v500}^{\#1} & G_{v500}^{\#2} & G_{v500}^{\#3} & 1 \end{array}\right]\left[\begin{array}{c} a_{1}\\ a_{2}\\ a_{3}\\ b \end{array}\right]$$ (4)

where *c*_j_^om^ was the simulated omics-guided weighting factors of dataset *j*; $G_{vj}^{\#1},\;G_{vj}^{\#2},\;G_{vj}^{\#3}$ were the transcriptomics data of three genetic markers, respectively, of dataset *j* from the validation dataset; and *a*_1_, *a*_2_, *a*_3_, *b* were the regression coefficients for the genetic markers and the linear part, respectively, which were determined in the “phenotype match” algorithm.

We then input these simulated omics-guided weighting factors into the dual-objective function to set up the omics-guided objective functions in a genome-scale metabolic model of *S*. *cerevisiae*, iND750:

$$\text{min}c_{j}^{om}\cdot \sum v_{i}^{2}-\left(1-c_{j}^{om}\right)\cdot v_{EtOH}$$ (5)

$$\begin{array}{l} \left[\begin{array}{c} s.t.\\ S\cdot v\;=\;0\\ lb\;\leq \;v_{i}\;\leq ub \end{array}\right]\\ c_{j}^{om}determined \end{array}$$ (6)

The ethanol yields could be predicted by solving this problem via the same quadratic solver, “*quadprog*”, in MATLAB. The flux ratios PGI/ZWF1, FBA/TKL, ENO/PCK, and PYK/PDC, were calculated to represent the key metabolic responses, i.e., glycolysis to oxidative pentose phosphate pathway (PP pathway), reductive PP pathway, futile cycle, and the fermentation pathway, respectively, in yeast metabolism. The variances of fluxes were obtained by flux variance analysis. The calculation of the variances of flux ratios were following the formula to calculate the combinational standard deviation [53].

$$R=\frac{F_{1}}{F_{2}},\;\frac{SD_{R}}{R}=\sqrt{{\left(\frac{SD_{F_{1}}}{F_{1}}\right)}^{2}+{\left(\frac{SD_{F_{2}}}{F_{2}}\right)}^{2}}$$ (7)

where, *R* is the key flux ratio, *F*_*1*_ and *F*_*2*_ are the flux values, *SD* is the standard deviation of the flux.

### Comparison of prediction accuracies and genetic markers of big data and omFBA algorithm

To evaluate the prediction accuracy of omFBA algorithm, we have compared the predicted ethanol yields to the observed ones from the validation dataset. We considered a prediction as a “matched” prediction if the relative error (*e*_*r*_) between the predicted and the observed ethanol yield was smaller 5%, as shown below:

$$e_{r}=\left|\frac{Y_{pred,j}-Y_{obs,vj}}{Y_{obs,vj}}\right|\times 100\%\leq 5\%$$ (8)

where, *Y*_pred,vj_ and *Y*_obs,vj_ are the predicted and observed ethanol yields (*w*/*w*) from the omFBA, respectively.

## Supporting Information

S1 Dataset MATLAB codes and input data to implement the omFBA and big data approaches. (ZIP)

S1 Fig Comparison of FBA prediction using dual objective function and the commonly used objective function. (TIF)

S2 Fig Correlation coefficient (absolute value) between gene expression and phenotype-matched weighting factors at low glucose condition and high glucose condition. (TIF)

S3 Fig Scheme for “Big Data” approach. In general, we directly correlated the transcriptomics and the phenotype data from the training dataset using regression analysis. Based on this regression equation derived, we applied the transcriptomics data from validation dataset to predict the phenotype, and compared the predictions and observations to evaluate the prediction accuracy. To start, we used the same training and validation datasets for big data approach as the ones we used for omFBA. We next calculated the absolute value of correlation coefficients between the transcriptomics data of each gene and the ethanol yields, and ranked them to find the top 3 genes with highest absolute values of correlation coefficients as the genetic markers. The transcriptomics data of these genetic markers have been used for multiple linear regressions to connect the gene expression with ethanol yield. Then, based on the regression equation, the ethanol yields were predicted from the transcriptomics data in validation dataset. Finally, the predicted ethanol yields were compared to the observed ethanol yields in the validation dataset. We repeated the “Big Data” regression approach for 40 times to make sure our predictions were statistically reliable.
(TIF)

S4 Fig Prediction accuracy of “Big Data” approach. Direct comparison of the predicted and observed ethanol yields in low (A) and high (B) glucose condition. The “Big Data” algorithm was repeated for 40 times and the proportions of matched predictions of “Big Data” algorithm were calculated and ranked for low (C) and high (D) glucose conditions.
(TIF)

S5 Fig Venn diagram for genetic markers identified by omFBA and “Big Data” using either “big pool” or “small pool” of genes for low and high concentration conditions. Top 10 genes with the highest absolute values of correlation coefficients were extracted from S1 Table and shown in this figure.
(TIF)

S6 Fig Correlation of phenotype-matched weighting factors derived from two genome-scale metabolic models of *S*. *cerevisiae*: iMM904 and iND750. (TIF)

S1 Table Function of genetic markers identified by omFBA and big data algorithms. (XLSX)

S2 Table Original datasets from iTAP database used in this study. (XLSX)
