# Supplementary figures and images for: OM-FBA: Integrate Transcriptomics Data with Flux Balance Analysis to Decipher the Cell Metabolism
Source: PLoS One. 2016 Apr 21;11(4):e0154188. doi: 10.1371/journal.pone.0154188 (PMC4839607; doi:10.1371/journal.pone.0154188)

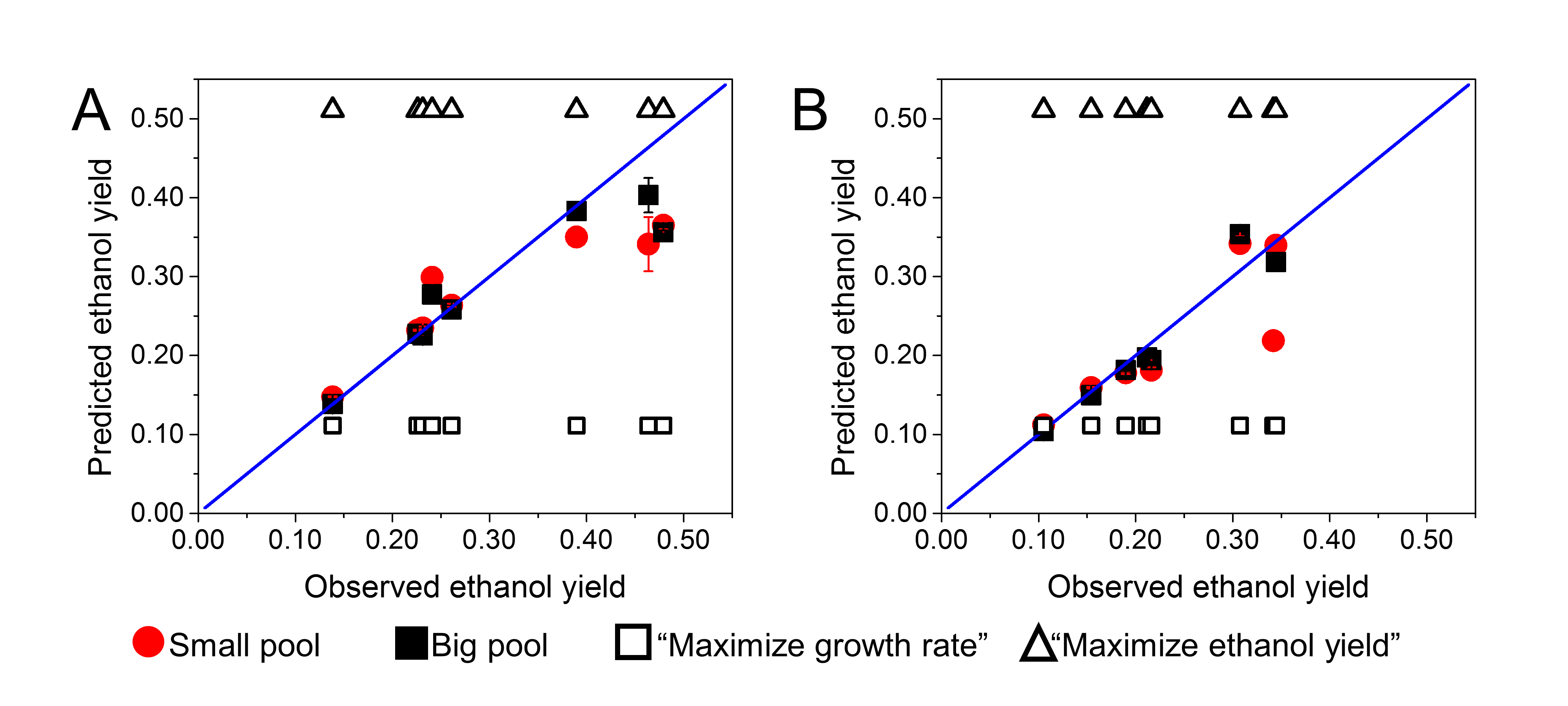

Supplement: S1 Fig — (TIF) [file pone.0154188.s002.tif]

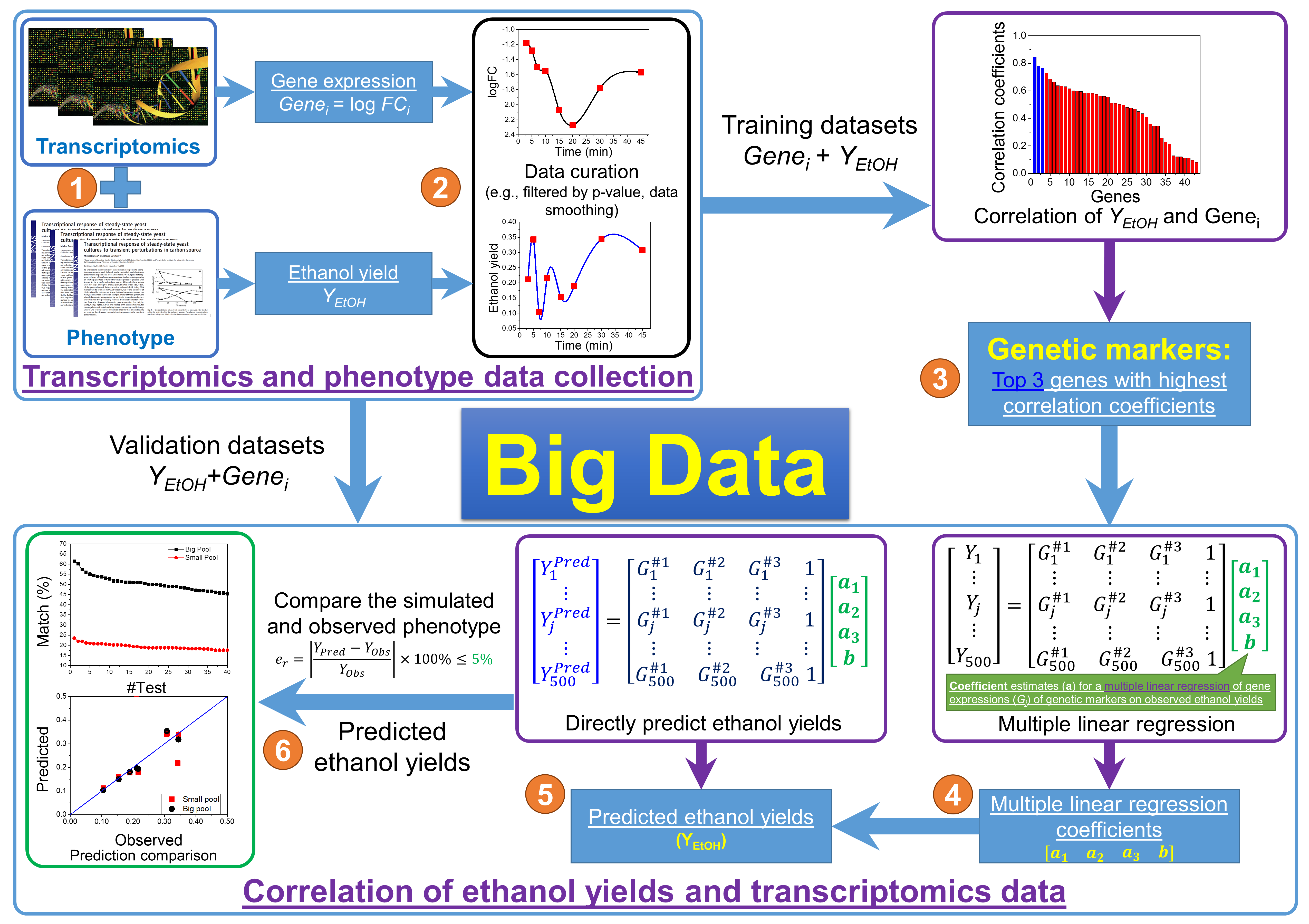

Supplement: S3 Fig — In general, we directly correlated the transcriptomics and the phenotype data from the training dataset using regression analysis. Based on this regression equation derived, we applied the transcriptomics data from validation dataset to predict the phenotype, and compared the predictions and observations to evaluate the prediction accuracy. To start, we used the same training and validation datasets for big data approach as the ones we used for omFBA. We next calculated the absolute value of correlation coefficients between the transcriptomics data of each gene and the ethanol yields, and ranked them to find the top 3 genes with highest absolute values of correlation coefficients as the genetic markers. The transcriptomics data of these genetic markers have been used for multiple linear regressions to connect the gene expression with ethanol yield. Then, based on the regression equation, the ethanol yields were predicted from the transcriptomics data in validation dataset. Finally, the predicted ethanol yields were compared to the observed ethanol yields in the validation dataset. We repeated the “Big Data” regression approach for 40 times to make sure our predictions were statistically reliable. (TIF) [file pone.0154188.s004.tif]

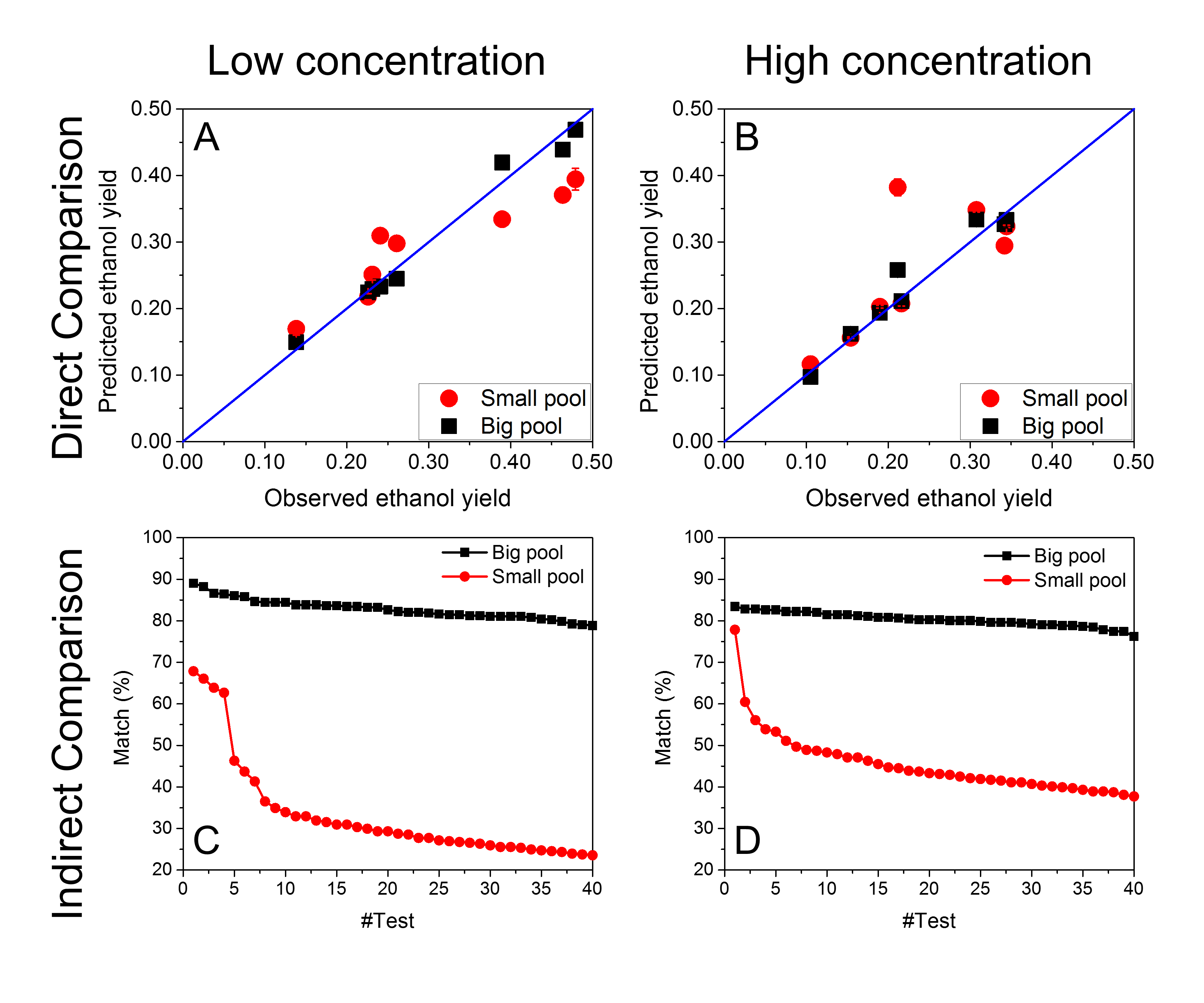

Supplement: S4 Fig — Direct comparison of the predicted and observed ethanol yields in low (A) and high (B) glucose condition. The “Big Data” algorithm was repeated for 40 times and the proportions of matched predictions of “Big Data” algorithm were calculated and ranked for low (C) and high (D) glucose conditions. (TIF) [file pone.0154188.s005.tif]

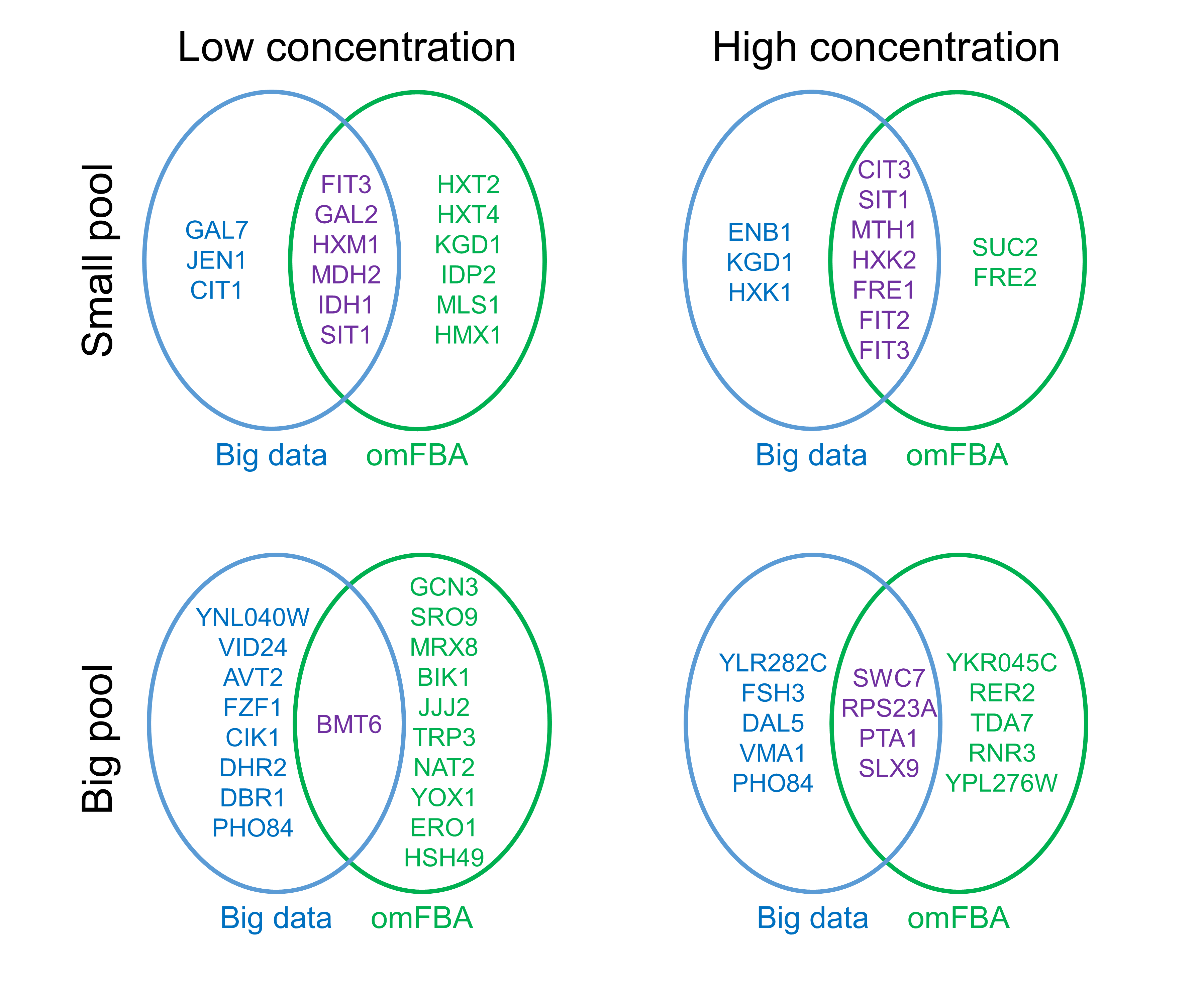

Supplement: S5 Fig — Top 10 genes with the highest absolute values of correlation coefficients were extracted from S1 Table and shown in this figure. (TIF) [file pone.0154188.s006.tif]
